# Supplementary material for: Early postnatal interactions between beige adipocytes and sympathetic neurites regulate innervation of subcutaneous fat
Source: eLife. 2021 Feb 16;10:e64693. doi: 10.7554/eLife.64693 (PMC7990502; doi:10.7554/eLife.64693)
Supplement: Supplementary file 1. [file elife-64693-supp1.docx]

Supplementary File 1. qPCR primer sequences used in this study

| Gene | Forward 5’-3’ | Reverse 5’-3’ |
| --- | --- | --- |
| *Ucp1* | ACT GCC ACA CCT CCA GTC ATT | CTT TGC CTC ACT CAG GAT TGG |
| *Cox8b* | GAA CCA TGA AGC CAA CGA CT | GCG AAG TTC ACA GTG GTT CC |
| *Cidea* | TGC TCT TCT GTA TCG CCC AGT | GCC GTG TTA AGG AAT CTG CTG |
| *Dio2* | CAG TGT GGT GCA CGT CTC CAA TC | TGA ACC AAA GTT GAC CAC CAG |
| *Elovl3* | TCC GCG TTC TCA TGT AGG TCT | GGA CCT GAT GCA ACC CTA TGA |
| *Prdm16* | GAC TTG GAC ACT ACC ACG GG | AGA TGC ACC CCC AAA CTC AG |
| *Ppargc1a* | CCC TGC CAT TGT TAA GAC C | TGC TGC TGT TCC TGT TTT C |
| *Cebpb* | TCG AAC CCG CGG ACT GCA AG | CGA CGA CGA CGT GGA CAG GC |
| *Fabp4* | ACA CCG AGA TTT CCT TCA AAC TG | CCA TCT AGG GTT ATG ATG CTC TTC A |
| *Pparg* | GTG CCA GTT TCG ATC CGT AGA | GGC CAG CAT CGT GTA GAT GA |
| *Adipoq* | GCA CTG GCA AGT TCT ACT GCA A | GTA GGT GAA GAG AAC GGC CTT GT |
| *S100b* | CAC CCG AAG AGG TTG CTC AT | GGA AGG GTG TAG GCG ATC AG |
| *Tgfb1* | GGA GAG CCC TGG ATA CCA ACT | TGT GTG TCC AGG CTC CAA AT |
| *Tbp* | GGGTATCTGCTGGCGGTTT | TGAAATAGTGATGCTGGGCACT |
